# Roles of Polymer Concentration and Ionic Strength in the Deposition of Chitosan of Fungal Origin onto Negatively Charged Surfaces

**DOI:** 10.3390/biomimetics9090534

**Published:** 2024-09-04

**Authors:** María Ormeño-Martínez, Eduardo Guzmán, Laura Fernández-Peña, Andrew J. Greaves, Lionel Bureau, Francisco Ortega, Ramón G. Rubio, Gustavo S. Luengo

**Affiliations:** 1 Departamento de Química Física, Facultad de Ciencias Químicas, Universidad Complutense de Madrid, Plaza de las Ciencias 2, Ciudad Universitaria, 28040 Madrid, Spain; mormeno@ucm.es (M.O.-M.); fortega@quim.ucm.es (F.O.); 2 Instituto Pluridisciplinar, Universidad Complutense de Madrid, Paseo Juan XXIII 1, 28040 Madrid, Spain; 3 Departamento de Tecnología Química, Energética y Mecánica, Escuela Superior de Ciencias Experimentales y Tecnología, Universidad Rey Juan Carlos, Calle Tulipán s/n, 28933 Móstoles, Spain; laura.fernandez.pena@urjc.es; 4 L’Oréal Research & Innovation,1 Avenue Eugène Schueller, 93600 Aulnay-sous-Bois, France; andrew.greaves@loreal.com; 5 Université Grenoble Alpes, CNRS, LIPhy, 38000 Grenoble, France; lionel.bureau@ujf-grenoble.fr

**Keywords:** adsorption, chitosan, cosmetics, ionic strength, solid surfaces

## Abstract

This study examines the potential of fungal chitosan derived from *Aspergillus niger* as a sustainable alternative to traditional petrochemical-based ingredients in cosmetic products. Specifically, the research examines the solubility of fungal chitosan in aqueous solutions of varying ionic strength and its adsorption onto negatively charged surfaces that mimic human hair keratin. The adsorption behavior, water content, and frictional properties of chitosan films were evaluated using a quartz crystal microbalance with dissipation monitoring and a surface force apparatus (SFA). The findings indicated that fungal chitosan exhibits good solubility at a pH of 4.5. Conversely, the adsorption of chitosan is subject to the influence of both polymer concentration and ionic strength. At the lowest ionic strengths, a screening-enhanced adsorption process occurs as a consequence of the reduction in chitosan solubility in the presence of salt. This results in the depletion of polymer chains from the solution and their subsequent deposition. An increase in ionic strength above 15–20 mM results in a worsening of the chitosan–surface interaction, due to the simultaneous screening of both the chitosan and the surface charges. This results in a hindrance to the adsorption process. The deposited films are highly hydrated, and this hydration increases with both polymer concentration and ionic strength. Furthermore, the films exhibit a predominantly elastic behavior, and the response of the films under shear deformations shows a strong dependence on the polymer concentration. These findings contribute to the development of environmentally friendly cosmetic formulations that meet consumer demands for sustainability.

## 1. Introduction

Over the last two decades, there has been a growing emphasis on reducing the potential risks and hazards to the environment associated with the use of non-renewable and non-biodegradable raw materials in consumer products. This has led to extensive research aimed at developing sustainable solutions [1]. This challenge is not straightforward. It necessitates a comprehensive reevaluation of the entire lifecycle of products and processes—from design and manufacturing to commercialization, use, and waste management. This is the so-called circular economy challenge [2,3,4]. The cosmetic sector is at the forefront of addressing environmental concerns associated with its processes and products. However, it is insufficient to merely adopt eco-friendly ingredients; innovations in packaging, production methods, end-of-life disposal, low carbon footprint and many other areas are also essential. By adopting a comprehensive approach, the industry can minimize its ecological footprint and promote a healthier interaction between consumer products and the environment [5,6,7,8].

In general, current cosmetic products comprise highly complex mixtures of ingredients, a significant proportion of which are derived from petrochemical sources. This reliance on petrochemicals necessitates a substantial investment in research, development, and innovation to identify and develop suitable alternatives. The substitution of these conventional ingredients is imperative for the enhancement of the sustainability of cosmetic products. In this context, one significant area of interest is the progressive substitution of cationic polymers, which are commonly used in shampoos and other hair care and conditioning products [9]. These conventional polymers are being replaced with eco-friendly and biodegradable alternatives that can be obtained from natural renewable materials [10]. This transition not only reduces the environmental impact, but also aligns with the growing consumer demand for sustainable and safe beauty products [11,12]. Chitosan, a naturally derived polysaccharide that can be obtained from diverse natural sources, including the exoskeleton of crustaceans and insects, or the cell walls of various species of fungi and algae, has emerged as a promising alternative in this context [13,14,15,16,17,18,19]. It possesses unique properties, including biocompatibility, biodegradability, and the capacity to form films and gels, which render it suitable for a wide range of cosmetic applications [20,21]. It is noteworthy that the formation of films resulting from the deposition of conditioning polymers, such as chitosan, on the surface of hair fibers does not provoke any biological processes. These films contribute solely to the temporary physical repair of the hair fiber surface, enhancing its appearance and texture without affecting the hair’s underlying biological structure. Consequently, while the hair may feel smoother and more manageable after treatment with these polymers, the effect is purely superficial and temporary, necessitating regular application to maintain the desired results.

The incorporation of chitosan into hair care products has the potential to enhance their conditioning and moisturizing properties while simultaneously contributing to their environmental sustainability [22,23]. Furthermore, the antimicrobial and anti-inflammatory properties of chitosan may confer additional benefits to the formulation, potentially enhancing the functionality of cosmetic products beyond just hair care [24,25,26,27,28]. Its use could extend to skincare formulations, where it might help in improving skin health and protection [29]. The renewed interest in the physico-chemical behavior of natural polysaccharides, such as chitosan, is indicative of a broader commitment on the part of the industry to innovation and sustainability [30,31].

This study examines the solubility of chitosan in aqueous solutions with varying ionic strengths and the deposition of chitosan from such solutions onto negatively charged surfaces that mimic key physico-chemical properties of mammalian keratin fibers, including surface charge and wettability, which are characteristic of human hair. Furthermore, the tribological properties of the resulting layers were evaluated using a Surface Force Apparatus (SFA), and the friction coefficient of fungal chitosan thin films was determined. This provides insights into the potential performance of these layers in hair care cosmetic applications. While the adsorption of chitosan on solid surfaces has been previously studied in the literature [32,33,34,35,36,37], this work represents the first attempt to study the adsorption of fungal chitosan. This is important because the utilization of chitosan derived from conventional crustacean sources is not the best choice for cosmetic applications due to several factors, including immunological concerns associated with residual proteins. The molecular weight and degree of deacetylation of chitosan can vary significantly depending on the source of the chitin and the subsequent hydrolysis process. These variations are particularly notable between crustacean and fungal chitosan. Fungal chitosan tends to have a much lower average molecular weight and polydispersity. It is also important to note that residual chitins can potentially pose immunological issues; therefore, complete hydrolysis is imperative. It is therefore crucial to gain a deep understanding of the behavior of chitosan derived from cosmetically acceptable sources, such as fungi, for the development of sustainable and safe beauty products. By exploring the unique properties of fungal chitosan, this research addresses both scientific and practical gaps in current knowledge. This study makes a significant contribution to scientific knowledge by providing detailed insights into various aspects of chitosan that are critical for its effective application in hair care products. Furthermore, the findings encourage the creation of environmentally friendly cosmetic solutions that align with the growing consumer demand for products that are not only effective, but also sustainable and safe. The research underscores the importance of sustainable raw materials in reducing the environmental footprint of cosmetic products and highlights the potential of fungal chitosan to revolutionize the industry by offering a viable, environmentally friendly alternative to traditional polymers. This work thus represents a significant contribution to ongoing efforts to innovate and improve the sustainability of cosmetic products, reflecting a broader commitment to environmental responsibility.

## 2. Materials and Methods

### 2.1. Chemicals

Chitosan from *Aspergillus niger* with an average molecular weight of 29.7 kDa and deacetylation degree of 97%, according to the supplier information, was supplied by PolymerExpert (Pessac, France). Sodium chloride (purity > 99.9%) was purchased from Merck (Darmstadt, Germany), and sodium hydroxide (purity 98.5%) and acetic acid, glacial (purity > 99.7%), were supplied by Fisher Scientific (Hampton, NH, USA).

The solutions were prepared using ultrapure deionized water of Milli-Q quality obtained using an AquaMAX™-Ultra 370 Series multi-cartridge purification system from Young Lin Instrument Co., Ltd. (Gyeonggi-do, Republic of Korea). This water exhibits a resistivity > 18 MΩ∙cm and a total organic content of <6 ppm.

### 2.2. Chitosan Solution Preparation

A stock solution of chitosan at a concentration of 5 g/L (or lower, depending on the maximum solubility at the specific considered ionic strength conditions) was prepared by weighing and pouring the necessary amounts of chitosan and NaCl into a 50 mL flask to obtain a stock solution with the desired composition. Subsequently, the flask was partially filled with water. Afterwards, 100 μL of glacial acetic acid was added to the flask to reduce the pH and ensure the complete solubilization of chitosan. The pH of the resulting chitosan solution was adjusted to 4.5 by the gradual addition of a 10^−2^ mM sodium hydroxide solution. Finally, the volume of the solution was adjusted to its target value by the addition of the required volume of a dilute aqueous acetic acid solution at pH = 4.5. To obtain solutions with lower concentrations, the required amount of stock solution was diluted with an aqueous acetic acid solution of pH = 4.5 and of the same ionic strength as the stock chitosan solution. The solubility of chitosan in aqueous mediums at pH = 4.5 and different ionic strengths was assessed directly by observing the presence of floating or sedimented solid particles in the aqueous solution. It should be noted that pH and ionic strength were fixed at values in the range of commercial shampoos and close to those of the scalp.

### 2.3. Methods

A QCM-Z500 quartz crystal microbalance with dissipation monitoring (QCM-D) from KSV Instruments Ltd. (Espoo, Finland), fitted with gold-coated AT-cut quartz crystals, was used to study the adsorption behavior of chitosan on negatively charged surfaces that simulate the characteristics of damaged hair. The clean quartz sensors were prepared by modifying their surfaces. This involves immersing the sensors in a *Piranha* solution (70% sulfuric acid/30% hydrogen peroxide) for 30 min, followed by thorough rinsing with Milli-Q water. Subsequently, a self-assembled monolayer of 3-mercapto-1-propane sulfonic acid (CAS No. 49594-30-1) was fabricated on the sensor surfaces. The formation of the self-assembled monolayer provides a permanent negative charge on the gold substrate of similar magnitude to that of damaged hair fiber. The QCM-D technique measures the impedance spectrum of a quartz crystal at its fundamental frequency (*f*_0_ = 5 MHz) and at odd harmonics up to the 11th overtone (central frequency, *f*_11_ = 55 MHz). The impedance spectra obtained were analyzed using a single-layer model following the methodology proposed by Voinova et al. [38], which correlates the changes in resonance frequency (Δ*f*) and dissipation factor (Δ*D*) across different overtones with the physical properties of the layers, including thickness, density, elasticity, and viscosity. For a detailed description of the data analysis procedure, please refer to our previous publication [39]. It is worth noting that the inclusion of the viscoelastic character of the film in QCM-D modeling, as opposed to the use of rigid boundary conditions, is essential and not arbitrary. Individual polymer layers typically adsorb onto surfaces in a manner that results in heterogeneous coverage. This heterogeneity cannot be accurately represented by assuming a rigid film, as has been extensively demonstrated in the literature [40,41,42]. In addition, the high degree of hydration commonly observed in polyelectrolyte layers introduces an additional dynamic component to the motion of the film. This component can only be adequately modeled by incorporating a viscous delay into the mechanical behavior of the film [40,43,44,45,46]. Thus, accounting for the viscoelastic nature of the film allows for a more accurate representation of its mechanical properties and interactions with the substrate, leading to a better understanding of its performance and stability in various applications.

An imaging null-ellipsometer (model EP^3^) from Nanofilm (Göttingen, Germany) was employed to quantify the optical thickness (*h*_op_) of the adsorbed layers on negatively charged solid surfaces. Ellipsometry experiments were conducted using a solid–liquid cell at a fixed angle of 60° with silicon wafers (Siltronix, Archamps, France) as substrates. The substrates were treated with *Piranha* solution for 30 min to generate a charged surface analogous to that of thiol-decorated gold surfaces [45]. During the ellipsometry experiments, the ellipsometric angles, *Δ* and *Ψ*, which are related to the ratio of the reflection coefficients for the parallel (*r*_p_) and normal (*r*_s_) components of the magnetic field, i.e., the ellipticity $\rho^{e}=\frac{r_{p}}{r_{s}}=e^{i\Delta }\tan\Psi$, were measured [47,48]. The optical thickness (*h*_op_) and refractive index (*n*) of the adsorbed layers are derived from these measurements using a slab model to describe the system. In this study, a four-layer slab model was employed, wherein the first layer represented the silicon substrate with a refractive index of *n*_Si_ = 4.1653 − 0.049i, and the second layer was the native oxide layer with a refractive index of *n*_SiO2_ = 1.4653. The thickness of the oxide layer was determined by measuring the bare silicon wafer in water. The fourth (outermost) layer of the model accounts for the solution surrounding the substate with a refractive index similar to that of the water (*n* = 1.33), as the polymer solutions are relatively diluted. The third layer was the adsorption layer. The thickness and refractive index of the adsorption layers were obtained by minimizing the differences between the experimental ellipsometric angles and the values calculated using Fresnel’s equations for the four-layer model [49].

It is important to note that the calculated values for *h*_ac_ (acoustic thickness from QCM-D measurements) and *h*_op_ should not be considered as absolute thicknesses, given the lateral heterogeneity present in most polyelectrolyte layers. Consequently, this study considers *h*_ac_ and *h*_op_ as effective thicknesses that provide different insights into the amount of material adsorbed within the layer. The combination of ellipsometry and QCM-D is crucial due to their distinct sensitivities to water. QCM-D quantifies the total mass of the adsorbed layer, including both the polymer and the associated water. In contrast, ellipsometry, which relies on the differences in refractive indices between the layer and the surrounding medium, measures solely the amount of adsorbed polymer. This yields *h*_op_ < *h*_ac_, enabling the estimation of the water content within the layers, according to the following expression [50,51],

(1) $$x_{w}=\frac{h_{ac}-h_{op}}{h_{ac}}.$$

The frictional properties of the adsorbed films were evaluated through shear experiments using a custom-built Surface Force Apparatus (SFA), as described by Bureau [52]. In these experiments, two freshly cleaved mica sheets, which had a negative surface charge, were attached to cylindrical lenses with a radius of *R* = 1 cm. The cylindrical lenses were then mounted in the SFA with their axes crossed at right angles, thereby creating a sphere/flat contact geometry. The chitosan was allowed to adsorb onto the mica surfaces from the surrounding solution for a period of 15 min. Following adsorption under quiescent conditions, the polymer solution was replaced with fresh solvent. Subsequently, three different types of experiments were conducted: (1) the normal force versus separation distance curve was determined by approaching and retracting the piezo at a velocity of 1 nm/s; (2) the friction forces were determined under normal loads ranging from 100 to 3500 μN at a constant shear speed of 10 μm/s; and (3) the dependence of friction on velocity was evaluated across a velocity range of 0.1 to 10 μm/s under a normal load of 1000 μN. The experiments yielded detailed insights into the frictional behavior of adsorbed polymer films under varying conditions.

## 3. Results

### 3.1. Chitosan Solubility in Water at pH = 4.5: Concentration and Ionic Strength Influence

One of the main challenges associated with the utilization of chitosan is its low solubility, which requires the optimization of the solubilization protocol and conditions [53,54]. A comprehensive understanding of the solubility range of chitosan is a crucial preliminary step in the optimization of chitosan-based formulations. This understanding allows the study of the adsorption process to be limited exclusively to conditions where chitosan is completely solubilized. Furthermore, chitosan solubility is important in the context of chitosan in commercial shampoo formulations, as the resolubilization of chitosan in the mixture after dilution of a shampoo can be a time-consuming process. Additionally, in the case of creams, chitosan is advantageous due to its capacity to form homogeneous films, which may be impeded in the presence of solid chitosan particles.

The analysis of chitosan solubility demonstrated that the fungal chitosan used in this study exhibits good solubility in water at pH = 4.5. It was observed that the solubility of the polymer was reduced in solutions with high polymer content (>4 g/L) and high ionic strength (100 mM). In these conditions, a sedimented solid phase was observed at the bottom of the aqueous phase. The high solubility of the fungal chitosan can be attributed to its high degree of deacetylation (>95%), which results in a relatively high positive charge density in mildly acidic conditions. Moreover, the low molecular weight of the chitosan used also plays a very critical role in its solubility. Indeed, the solubility profile of the chitosan studied here is significantly better than that of the fungal chitosan with a higher molecular weight (133.3 kDa) and similar degree of deacetylation. Specifically, for the higher molecular weight chitosan, the maximum solubility was found to be reduced to 4 g/L at 40 mM ionic strength and further reduced to 3 g/L at 100 mM. This indicates that as the molecular weight of chitosan increases, its solubility in aqueous solutions decreases, especially at higher ionic strengths, highlighting the importance of using lower molecular weight chitosan for optimal solubility in cosmetic formulations [55].

The reduction in solubility at high polymer concentrations and high ionic strengths can be attributed to the worsening in the quality of water as a solvent for chitosan under such conditions. Consequently, the low molecular weight of the polymer results in a low number of monomers per chain, which facilitates charge shielding upon salt addition and thus, its separation as a solid phase [56].

### 3.2. Effect of Solution Concentration on the Adsorption of Chitosan onto a Negatively Charged Surface

**Figure 1:**
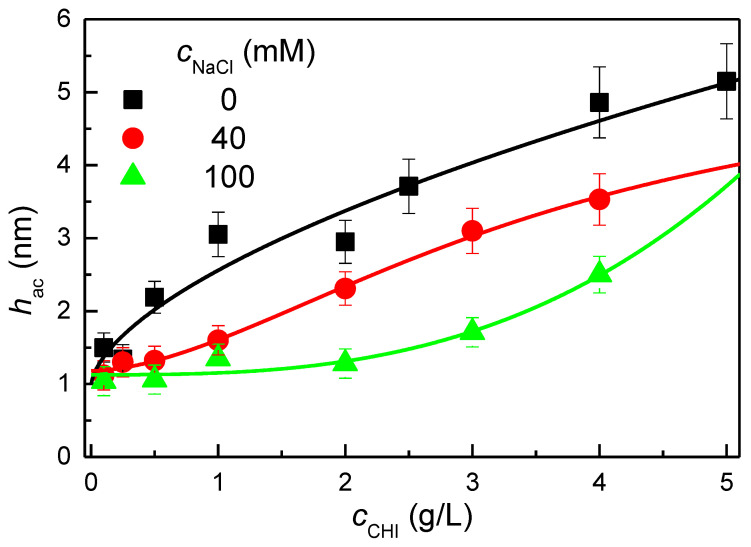
Dependence of the acoustic thickness, *h*_ac_, on the concentration of chitosan in the solution, *c*_CHI_, for chitosan deposition from solutions with different ionic strengths. The lines are only guides for the eyes.

One of the main aspects that allows the modulation of the deposition of polycations such as chitosan onto solid surfaces is the concentration of the solution [32]. By adjusting the concentration of chitosan in the solution, it may be possible to fine-tune the thickness and uniformity of the resulting polymer layer. This property is particularly significant in various applications where precise control over the film characteristics is crucial. The cosmetic industry can leverage this capability for developing advanced formulations and products. Figure 1 shows the dependence of the acoustic thickness, *h*_ac_, obtained with a QCM-D, on the concentration of chitosan in solution, $c_{CHI}$, for the deposition of chitosan layers from polymer solutions with different ionic strengths. It is important to emphasize that the results presented correspond to acoustic thicknesses measured after rinsing with an aqueous solution that matches the pH and ionic strength of the solution used for deposition. The discussion of thickness after rinsing is critical when dealing with conditioning depositions because the final conditioning layer is typically formed after a rinsing process that mimics the practical conditions often encountered during hair shampooing and conditioning. The rinse step is particularly important because it allows for the removal of loosely bound molecules or excess material, leaving a more stable and representative layer. Therefore, the acoustic thickness measured after rinsing provides a more accurate reflection of the properties of the final layer, ensuring that the data correspond to the actual functional layer that will be present under real-world conditions. A model that accounts for the viscoelastic properties of the deposited films was used to calculate the acoustic thickness data. This approach was necessary because the frequency and dissipation shifts observed for different overtones of the quartz crystal did not overlap, either during the adsorption process or during the rinsing step. The lack of overlap indicates that the layers exhibit significant viscoelastic behavior, which affects how they interact with the oscillating quartz crystal.

The results show a clear trend where the acoustic thickness increases with chitosan concentration, as would be expected for the adsorption isotherm of a polymer on a solid surface [40]. This can be understood by considering that an increase in the polymer concentration results in an increase in the number of available charged groups as the number of polymer chains increases, i.e., the number of points used for the polymer to interact with the surface increases. This increases the competition between the polymer chains for the adsorption sites on the solid surface. Consequently, the adsorption of the chains would occur by forming layers with a high number of chains bounded by a reduced number of points. This results in the formation of layers comprising an increased number of segments protruding into the solution, leading to an overall increase in the average layer thickness [57]. The concentration dependence of the adsorbed amount demonstrates the potential for regulating the deposition process through simple concentration adjustments. On the other hand, the analysis of the adsorption results for solutions with different ionic strengths indicates that the dependence of the amount adsorbed on the polymer concentration is analogous regardless of the NaCl concentration in the solution. Nevertheless, a detailed analysis of the impact of salt concentration on adsorption shows that the thickness of the layers initially increases with ionic strength and then decreases as the ionic strength of the solution is further increased. However, this dependence deserves further analysis, which will be carried out in the following section.

### 3.3. Effect of Solution Ionic Strength on the Adsorption of Chitosan onto a Negatively Charged Surface

**Figure 2:**
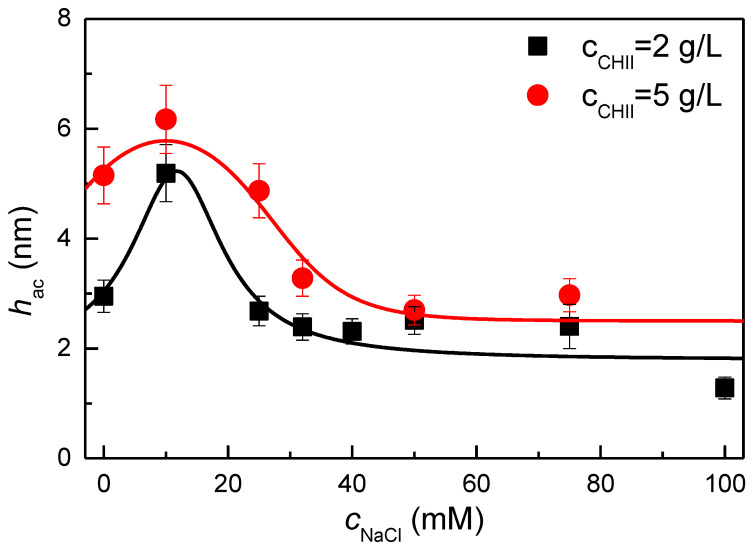
Dependence of the acoustic thickness, *h*_ac_, on the NaCl concentration in the solution, *c*_NaCl_, for the deposition of chitosan from solutions with concentrations of 2 and 5 g/L. The lines are guides for the eyes.

As shown in Section 3.2, the ionic strength exerts a profound impact on the amount of polyelectrolyte (chitosan) adsorbed onto the solid surface. Figure 2 shows the dependence of the acoustic thickness of the deposited layer on the NaCl concentration, $c_{NaCl}$, for chitosan solutions with two different selected polymer concentrations (2 and 5 g/L).

As previously indicated by the results presented in Figure 1, the adsorption process appears markedly influenced by the NaCl concentration in the solution. Figure 2 illustrates the emergence of two distinct regimes for adsorption as the ionic strength of the polymer solutions increases. This behavior can be explained by considering the charge screening of the polymer chains and the surface. At low ionic strengths, below a NaCl concentration of approximately 15–20 mM, an increase in the thickness of the deposited film is observed as the ionic strength increases, irrespective of the polymer concentration. This is the typical behavior expected for the adsorption of the majority of polycations onto negatively charged surfaces and can be explained by considering the role of the salt in shielding electrostatic interactions [49].

The shielding of the chitosan charges by the salt ions causes a transition in the conformation of the polymer from a rod-shaped conformation to a more coiled conformation. Moreover, it worsens the quality of water as a solvent for the polyelectrolyte chains. This favors the separation of the polyelectrolyte chains from the solution, which in turn facilitates their deposition on the solid surface, thereby increasing the layer thickness. Furthermore, the conformational transition of the polymer chains results in the deposition of layers with segments protruding into the solution, which contributes to the observed increase in layer thickness with increasing ionic strength.

As the ionic strength exceeds a threshold value, approximately at 15–20 mM, the thickness begins to decrease with increasing ionic strength, resulting in anti-polyelectrolyte behavior [58]. This is due to the fact that the addition of NaCl not only shields the charge of the polyelectrolyte, but also that of the solid surface. At this point, the complex interplay between the enhanced polyelectrolyte adsorption resulting from charge shielding and the shielding of the surface will determine the final adsorption result [59]. Therefore, based on the aforementioned results, it can be concluded that the adsorption of the polyelectrolyte implies a competition between salt ions and the polymer chains for the available sites on the surface. At high ionic strengths, the number of available sites on the surface is significantly reduced due to the adsorption of salt ions. Moreover, the shielding of the charges of the polymer chains also results in a reduction in their effective charge. This double shielding effect results in a reduction in the probability of electrostatic interactions between the surface and the chitosan, which in turn leads to a reduction in the overall thickness of the layers. Based on the results, it is possible to define two distinct regimes for the adsorption of chitosan onto oppositely charged surfaces: (i) at low ionic strength, an enhanced adsorption by charge screening occurs (typical polyelectrolyte behavior), and (ii) at high ionic strength, a worsened adsorption by charge screening occurs (anti-polyelectrolyte behavior). It should be noted that the change in the adsorption regime with the ionic strength is compatible with the change in the role of entropic contribution to the adsorption process. In fact, when the ionic strength is negligible or very low, the release of counterions occurring as a result of the ionic pairing between the chitosan chain and the surface results in a noticeable increase in the entropy of the system. On the contrary, when the ionic strength increases, the entropic gain as a result of the counterion release is reduced, and consequently, a high proportion of the counterions tend to remain attached to the chitosan and the surface, which hinders the direct electrostatic interaction between the polyelectrolyte chains and the surface. Therefore, the two regimes of adsorption are equivalent to consider two different regimes of charge compensation in the adsorbed layers. At low ionic strength, the charge compensation occurs by direct electrostatic pairing between chitosan and the surface, which results in an intrinsic compensation mechanism. On the contrary, as the ionic strength increases, the role of the direct electrostatic interaction between the polymer and the surface is reduced, and the compensation becomes mainly extrinsic, where the counterions play an essential role in the charge neutrality.

### 3.4. Water Content of the Layers: A QCM-D and Ellipsometry Study

**Figure 3:**
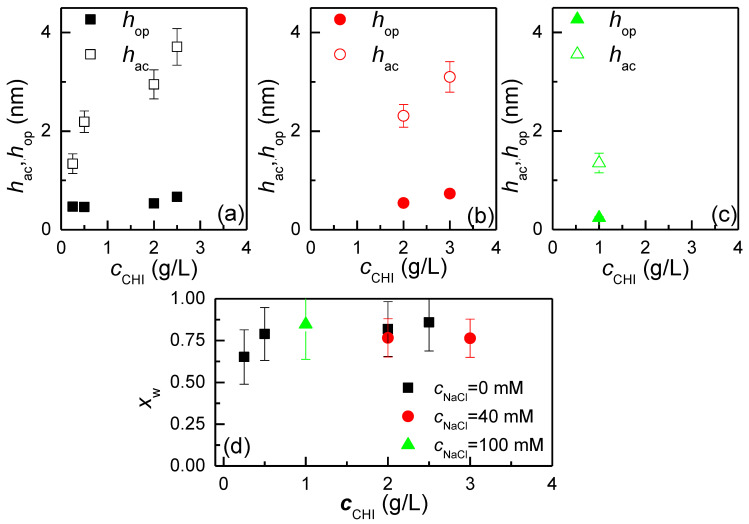
(**a**) Dependences of the acoustic and optical thicknesses on the chitosan concentration for the deposition of layers from solutions with a NaCl concentration of 0 mM. (**b**) Dependences of the acoustic and optical thicknesses on the chitosan concentration for the deposition of layers from solutions with a NaCl concentration of 40 mM. (**c**) Dependences of the acoustic and optical thicknesses on the chitosan concentration for the deposition of layers from solutions with a NaCl concentration of 1000 mM. (**d**) Dependences of the water content on the chitosan concentration for layers obtained by deposition from solutions with different NaCl concentrations.

An important parameter in the characterization of films of cosmetic interest is the quantification of the amount of water associated with them. This can be achieved by comparing the optical thickness values obtained through ellipsometry, *h*_op_, and the acoustic thickness value, *h*_ac_, obtained through QCM-D (see Equation (1)). Figure 3 shows the differences between the optical and acoustic thicknesses obtained for the deposition of chitosan layers from solutions with different polymer concentrations and ionic strengths.

**Figure 4:**
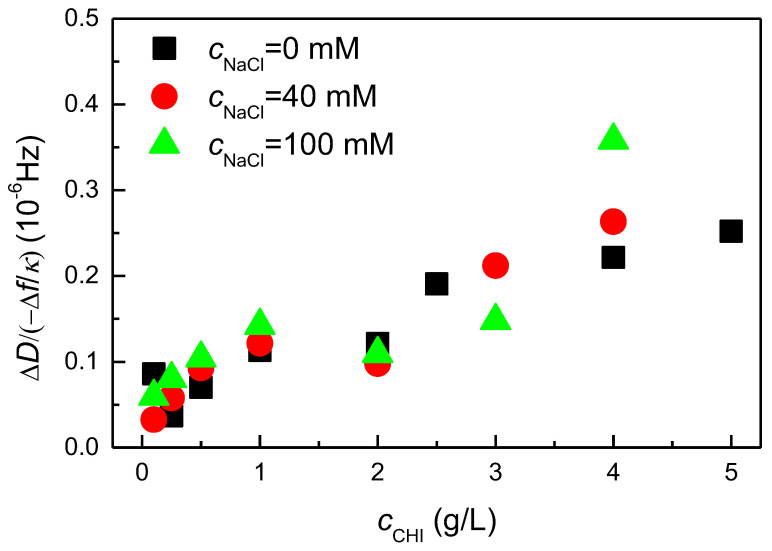
Dependence of −Δ*D*/Δ*f* on chitosan concentration for the deposition of chitosan layers from aqueous solutions with different ionic strength. Note that the frequency is normalized by the overtone number, in this case the third (*κ* = 3).

The results show that the optical thickness values are always lower than the acoustic thickness (*h*_op_ < *h*_ac_), indicating that the actual content of chitosan in the deposited layers is relatively low. Consequently. it can be anticipated that the formed films will exhibit a high degree of hydration. The high degree of hydration is evident from the results shown in Figure 3d, where it is observed that the hydration of the films, obtained by Equation (1), slightly increases with the concentration of the polymer, whereas the effect of the ionic strength of the solutions appears less important. This is consistent with the formation of layers with an elevated number of segments oriented towards the solution, which can trap a high amount of water. It is crucial to highlight that the formation of layers with a high number of segments protruding into the solution is expected to correlate with an increase in oscillation resistance in quartz crystal microbalance experiments. This is expected to contribute to an increase in the dissipation factor. This can be evaluated in terms of the −Δ*D*/Δ*f* ratio obtained in the QCM-D experiments. Figure 4 shows the dependence of the −Δ*D*/Δ*f* ratio, where the −Δ*f* appears divided by the number of overtone *κ* (−Δ*f*/*κ*) which assumes a value of 3, on the chitosan concentration for the deposition from solutions without added NaCl. An increase in the −Δ*D*/Δ*f* ratio indicates a higher energy dissipation during the oscillation, thereby confirming the formation of more diffuse layers, i.e., with a higher number of segments oriented towards the solution.

### 3.5. Shear Mechanical Response of the Adsorbed Layers

**Figure 5:**
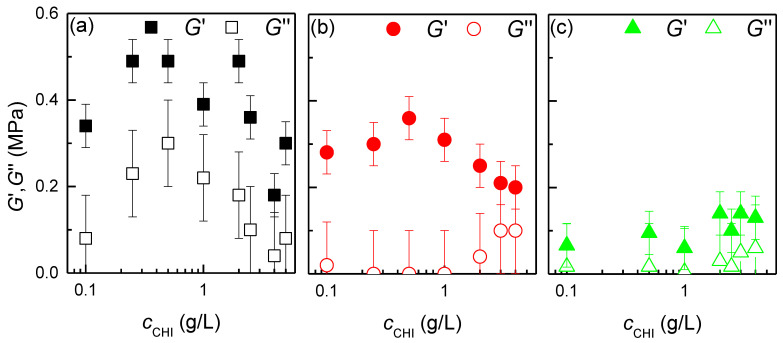
Dependence of the real (*G*’, solid symbols) and imaginary (G″, open symbols) components of the viscoelastic shear modulus on the concentration for chitosan layers obtained from aqueous solutions with different ionic strength. (**a**) *c*_NaCl_ = 0 mM. (**b**) *c*_NaCl_ = 40 mM. (**c**) *c*_NaCl_ = 100 mM.

QCM-D also provides insight into the mechanical properties of the deposited films, in the form of the real and imaginary components of the viscoelastic shear modulus, i.e., G′ and G″. Figure 5 shows the dependences of the two components of the viscoelastic shear modulus on the chitosan concentration, which were obtained for films deposited from chitosan solutions of different ionic strengths.

The results show that the real and imaginary components of the viscoelastic shear modulus exhibit a value of the same order across all studied conditions, which can be explained by the fact that the obtained layers present a rubber-like character. This aligns with the plasticizing effect of the water. Nevertheless, a more detailed analysis reveals that, across all investigated scenarios, the elastic component has a greater importance (*G*′ > *G*″). Additionally, while *G*″ exhibits minimal variation with the adsorption conditions, remaining always within the range 0.05–0.1 MPa, *G*′ decreases with increasing ionic strength. This can be attributed to the fact that an increase in the ionic strength results in the formation of films with a fuzzier structure. Consequently, the importance of the elastic component over the viscous one is reduced due to a higher degree of hydration and swelling of the layers, which increases their deformability. On the other hand, an analysis of the dependence of the mechanical response of the layers on the variation of the polymer concentration reveals that at low polymer concentrations, the elastic component increases and then decreases. This can be attributed to the fact that at low concentrations, the amount of polymer is not sufficient to fully cover the surface, resulting in the formation of highly heterogeneous layers comprising polymer islands surrounded by water.

### 3.6. Frictional Properties of Chitosan Layers

**Figure 6:**
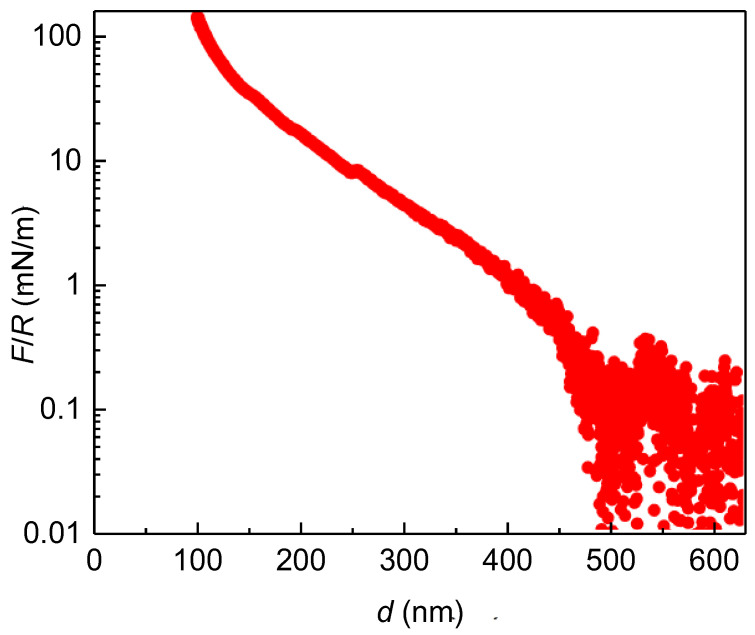
Normalized normal load vs. inter-surface distance curve (F/R curve) measured upon approaching the surfaces, for layers of chitosan obtained from the adsorption of a solution with concentration 5 g/L in pure water.

Controlling the tribological properties of a coating is critical, as the frictional properties of the film allow the regulation of stiction and lubrication on surfaces, which significantly affects the potential applications of the film [39,60,61]. This section will examine the similarities and differences in the frictional properties obtained from SFA experiments conducted on chitosan films obtained as a result of adsorption of solutions with different polymer concentrations and the same ionic strength. Figure 6 shows the normal force (*F*) vs. separation distance (*d*) curve obtained for a layer resulting from the adsorption of a chitosan solution of concentrations 5 g/L in water.

**Figure 7:**
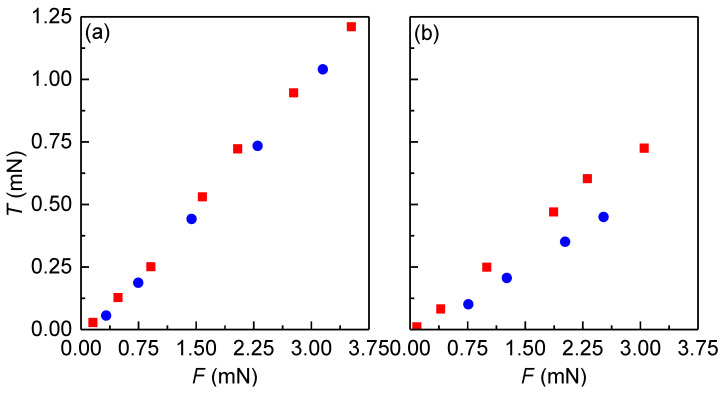
Friction force vs. normal load curves obtained at a fixed shear speed of 10 μm/s for chitosan layers deposited from solutions with different polymer concentrations without NaCl and at pH = 4.5: 5 g/L (**a**) and 2 g/L (**b**). In both panels: (■) increasing normal force and (●) decreasing normal force.

The results show that as the surfaces approach each other, a repulsive force emerges, exhibiting a pronounced increase in magnitude at distances below 500 nm. This repulsive force set in an abnormally large distance. Furthermore, the results indicate that even under the highest applied load, the surface separation is extremely high, with the layer trapped between the surfaces measuring approximately 100 nm. This can be only explained by considering the heterogeneity of the chitosan adsorption layers, which contain submicron aggregates that prevent a close contact between the surfaces. The presence of these aggregates makes it difficult to reliably evaluate normal interactions between surfaces. In fact, the sequential measurement of the approach/retract curves was not possible in these conditions, and hence it was not possible to evaluate the adhesion. Nevertheless, despite the inability to assess typical surface interactions, it was possible to characterize the frictional properties of the chitosan films. To this aim, the tribological properties of chitosan layers were evaluated at a fixed shear speed of 10 μm/s by measuring the frictional forces (*T*) as the applied normal frictional load (*F*) was increased within the range 100–3500 μN (*F*/*R* ratio from 10 to 230 mN/m). The representation of *T* vs. *F* curves under compression and expansion conditions for chitosan layers deposited from aqueous solutions of different concentrations is shown in Figure 7.

The frictional forces corresponding to the chitosan layers were found to be relatively high, with the frictional force (*T*) being proportional to the normal load (*F*) regardless of the composition of the layers, and the friction coefficient, defined by the slope of the representation (*μ* = *T*/*F*), being relatively high. However, the dependences of the friction vs. load curves under load and unload are strongly dependent on the layer composition. In fact, for films deposited from solutions with a chitosan concentration of 5 g/L, there is no hysteresis in the load–unload curves, resulting in a friction coefficient of 0.34 without any evidence of wear in the deposited film. On the other hand, when the chitosan concentration is reduced to 2 g/L, the appearance of hysteresis in the load–unload cycle was found. This suggests history-dependent frictional properties of the films, which can be explained by considering the heterogeneity of the chitosan layers due to the existence of a low coverage and the presence of sub-micron aggregates in their structure. This can result in layer reorganization under shear, which reduces the friction coefficient from 0.27 during the load process to 0.21 during the unload process. The data thus demonstrate the pivotal role of the chitosan concentration in regulating the lubrication properties of chitosan films, with implications for layer coverage and structure.

**Figure 8:**
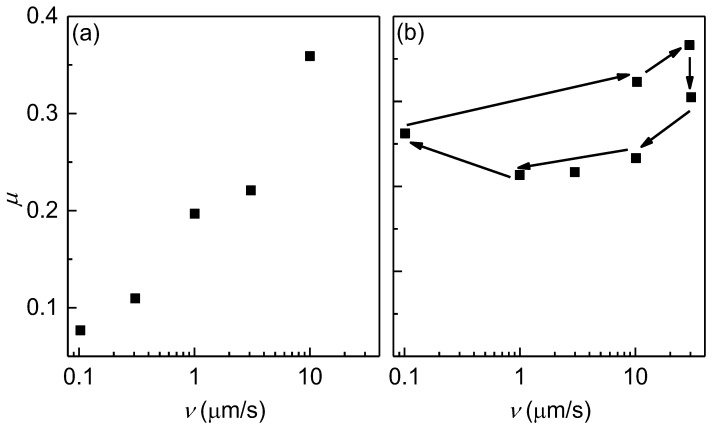
Dependence of the friction coefficient on the shear velocity at a fixed load of 1 mN/m for chitosan layers deposited from solutions with different polymer concentrations without NaCl and at pH = 4.5: 5 g/L (**a**) and 2 g/L (the arrows indicate the hysteresis cycles) (**b**).

The concentration-dependent frictional properties of the layers results in markedly different dependences of the friction coefficient (*μ*) on the shear velocity, *v* (see Figure 8). At a high chitosan concentration (5 g/L), a weak quasi-logarithmic increase in friction with the sliding speed was observed. Conversely, when the layers were deposited from a more diluted solution (2 g/L), a very complex dependence of the friction on the sliding speed was found, which may be attributed again to the layer heterogeneity.

## 4. Conclusions

This article presents several novel findings that enhance our understanding of the behavior and potential of fungal chitosan in cosmetic applications. In particular, the study shows the solubility profile of fungal chitosan at pH 4.5, revealing optimal solubility conditions that avoid the formation of sedimented solids, which is critical for its effective formulation in cosmetic products. The research shows that the high degree of deacetylation and low molecular weight of fungal chitosan significantly contribute to its solubility, which is essential for the development of stable cosmetic formulations. Moreover, this may contribute to enhance the efficiency and quality of cosmetic products, ensuring they remain smooth and free from sedimentation. Furthermore, by combining a pool of surface-sensitive experimental techniques, new insights into the adsorption behavior of fungal chitosan onto negatively charged surfaces have been obtained, highlighting the significant influence of polymer concentration and ionic strength on the formation and properties of the adsorbed layers. The results have shown that the deposition of the chitosan on the negatively charged surfaces is the result of an intricate balance of different electrostatic interactions occurring within the system. In fact, the charge screening of both the chitosan chains and the surface, as a result of changing the ionic strength of the solution, allows tailoring the chitosan deposition. This knowledge is essential for optimizing chitosan-based formulations for hair care and other cosmetic applications. Two distinct regimes, namely polyelectrolyte and anti-polyelectrolyte, of adsorption were found depending on the ionic strength of the solutions. Thus, at very low ionic strength (<20 mM), the deposition is enhanced with increasing ionic strength due to the worsening quality of the water as a solvent for the chitosan and the favored depletion of the polymer chains from the solution to the surface. This means that in the absence of salt, the ionizable groups on the polyelectrolyte remain highly solvated due to the polymer conformation and the minimal shielding by counterions. However, when salt is added, the introduced ions shield these charged groups, reducing their solvation. This shielding effect leads to a decrease in the solubility of the polyelectrolyte in water as the polymer chains become more compact and with a lower effective charge.

Increasing the ionic strength above a threshold concentration (around 20 mM) leads to a decrease in deposition due to the shielding of the negative charge of the surface, which limits an effective electrostatic interaction between chitosan and the surface. It is worth mentioning that the control of the deposition by the composition of the formulation is particularly relevant for hair care products, where controlled deposition of chitosan can enhance conditioning and protective effects. In addition to the adsorption of material, an important parameter in the characterization of polymer films with potential application in hair care cosmetics is the amount of water associated with them. The results indicate that there is a high degree of water associated with the polymer films. This hydration increases with polymer concentration and ionic strength due to an increase in the number of segments protruding into the solution, which is consistent with the information extracted from the dissipation values obtained from the QCM-D experiments. Thus, the increase in chitosan concentrations leads to more diffuse layers with higher energy dissipation. Furthermore, the viscoelastic shear modulus components showed a predominantly elastic nature of the layers (*G*′ > *G*″), with *G*′ decreasing with increasing ionic strength, suggesting more deformable, hydrated layers. This plays a crucial role in developing hydrating and flexible cosmetic films that can impact the effectiveness of conditioning formulations. On the other hand, the frictional properties varied with polymer concentration, with high-concentration films (5 g/L) exhibiting consistent friction coefficients without hysteresis, while lower-concentration films (2 g/L) were subject to shear wear due to structural heterogeneity and the presence of aggregates. These results highlight the critical role of the chitosan concentration and ionic strength in modulating film hydration, mechanical integrity, and tribological performance, which is essential for tailoring chitosan-based films for specific cosmetic applications. In fact, the results have demonstrated that by manipulating the chitosan concentration in formulations, manufacturers may create products with tailored properties, such as improved spreadability, enhanced sensory attributes, and prolonged wearability, meeting diverse consumer needs and preferences.

It is worth noting that films 1 to 5 nm thick, such as those obtained in this work, could be valid as coatings for hair conditioning, providing a lightweight and smooth layer that can improve hair texture and manageability. However, the heterogeneity and presence of sub-micrometric aggregates in these films could pose significant challenges. Uneven coating can lead to inconsistent conditioning effects, with some areas of the hair receiving more product than others, potentially causing uneven smoothing or weighing down of the hair. Additionally, these aggregates might lead to a rough texture or visible residue, detracting from the desired silky and uniform feel of conditioned hair. Thus, ensuring homogeneity and minimizing aggregates are crucial for the effective performance of such hair conditioning systems. Overall, this work provides substantial evidence for the practical applicability of fungal chitosan in cosmetics, paving the way for future research to explore its full potential. Researchers can build on these findings to develop more effective, sustainable and consumer-friendly cosmetic products.

## Data Availability

Data are available upon reasonable request.
